# PacBio assembly of a *Plasmodium knowlesi* genome sequence with Hi-C correction and manual annotation of the *SICAvar* gene family

**DOI:** 10.1017/S0031182017001329

**Published:** 2017-07-19

**Authors:** S. A. LAPP, J. A. GERALDO, J.-T. CHIEN, F. AY, S. B. PAKALA, G. BATUGEDARA, J. HUMPHREY, the MaHPIC consortium, J. D. DeBARRY, K. G. Le ROCH, M. R. GALINSKI, J. C. KISSINGER

**Affiliations:** 1 Emory Vaccine Center, Yerkes National Primate Research Center, Emory University, Atlanta, GA, USA; 2 Federal University of Minas Gerais, Belo Horizonte, MG, Brazil; 3 René Rachou Research Center (CPqRR-FIOCRUZ), Belo Horizonte, MG, Brazil; 4 Department of Mathematics and Computer Science, Emory University, Atlanta, GA, USA; 5 La Jolla Institute for Allergy and Immunology, La Jolla, CA 92037, USA; 6 Institute of Bioinformatics, University of Georgia, Athens, GA 30602, USA; 7 Center for Tropical and Emerging Global Diseases, University of Georgia, Athens, GA 30602, USA; 8 Center for Disease and Vector Research, Institute for Integrative Genome Biology, Department of Cell Biology & Neuroscience, University of California Riverside, CA 92521, USA; 9 Malaria Host-Pathogen Interaction Center, http://www.systemsbiology.emory.edu/; 10 Division of Infectious Diseases, Department of Medicine, Emory University, Atlanta, GA, USA; 11 Department of Genetics, University of Georgia, Athens, GA 30602, USA

**Keywords:** *Plasmodium knowlesi*, PacBio, Hi-C, *SICAvar*, MaHPIC, genome, sequence, annotation, antigenic variation

## Abstract

*Plasmodium knowlesi* has risen in importance as a zoonotic parasite that has been causing regular episodes of malaria throughout South East Asia. The *P. knowlesi* genome sequence generated in 2008 highlighted and confirmed many similarities and differences in *Plasmodium* species, including a global view of several multigene families, such as the large *SICAvar* multigene family encoding the variant antigens known as the schizont-infected cell agglutination proteins. However, repetitive DNA sequences are the bane of any genome project, and this and other *Plasmodium* genome projects have not been immune to the gaps, rearrangements and other pitfalls created by these genomic features. Today, long-read PacBio and chromatin conformation technologies are overcoming such obstacles. Here, based on the use of these technologies, we present a highly refined *de novo P. knowlesi* genome sequence of the Pk1(A+) clone. This sequence and annotation, referred to as the ‘MaHPIC Pk genome sequence’, includes manual annotation of the *SICAvar* gene family with 136 full-length members categorized as type I or II. This sequence provides a framework that will permit a better understanding of the *SICAvar* repertoire, selective pressures acting on this gene family and mechanisms of antigenic variation in this species and other pathogens.

## INTRODUCTION

*Plasmodium knowlesi* is recognized as a zoonotic parasite and widespread public health threat in South East Asia, with acute and severe illness requiring hospitalization (Singh *et al.* 2004; Singh and Daneshvar, 2013; Muller and Schlagenhauf, 2014; Ahmed and Cox-Singh, 2015; Wesolowski *et al.* 2015). There has been a natural, increasing interest and momentum towards understanding the genetic, biological and pathogenic mechanisms that operate during *P. knowlesi* infections both in humans and non-human primates (Cox-Singh and Culleton, 2015; Millar and Cox-Singh, 2015). To fight this zoonosis, there is a need to understand the geographical distribution of the parasite and its dynamics to enable local and global actions for rapid treatment and work to break the cycle of transmission (Moyes *et al.* 2014; Shearer *et al.* 2016; Barber *et al.* 2017).

*Plasmodium knowlesi* has served as a model parasite for malaria research for over 50 years, as detailed elsewhere in this special issue (Galinski *et al.* 2017; Pasini *et al.* 2017). Here we stress the importance and value of a well-assembled and annotated genome sequence for the continued benefit of many aspects of this research. An accurate reference genome sequence can be important for vaccine and drug target discovery, and critical for certain basic biology studies. This is especially the case for studying the evolution of complex multigene families, their regulation and antigenic variation. *Plasmodium knowlesi* has been instrumental in antigenic variation research since the discovery of this phenomenon in 1965 in the context of longitudinal *P. knowlesi* infections of *Macaca mulatta* (rhesus macaques) (Brown and Brown, 1965). A few additional landmark discoveries include the identification of variant antigen proteins in 1983, namely the schizont-infected cell agglutination (SICA) proteins (Howard *et al.* 1983), and the demonstration of the importance of the spleen for their expression and their association with virulence (Barnwell *et al.* 1983). The large multigene family that encodes the SICA proteins was published in 1999 and named *SICAvar* (al-Khedery *et al.* 1999; Pain *et al.* 2008; Lapp *et al.* 2009). The originally identified *SICAvar* gene, with large introns and 12 exons, revealed the complexity of individual gene family members (al-Khedery *et al.* 1999; Pain *et al.* 2008; Lapp *et al.* 2009).

While extremely valuable as an initial starting point, the first published *P. knowlesi* genome had, as most complex genome sequences do, many gaps, fragmented genes and misplaced sequences (Pain *et al.* 2008; Lapp *et al.* 2009). Here we utilize a combination of long-read Pacific Biosciences (PacBio) and High-throughput Chromosome Conformation Capture (Hi-C) technologies to develop a more complete *P. knowlesi* genome sequence and complement this with both automated and manual annotation. This effort includes a complete annotation of the *SICAvar* gene family, which now totals 117 type I and 19 type II genes. There are also 22 ‘*SICAvar*’ gene fragments that remain throughout the genome that predominantly contain the two 5′ most exons or the final three exons. This updated *P. knowlesi* genome sequence is referred to as the ‘MaHPIC Pk genome sequence’ since it was generated as a part of the Malaria Host-Pathogen Interaction Center (MaHPIC) project (http://systemsbiology.emory.edu).

## MATERIALS AND METHODS

### Parasite strain and culture

The MaHPIC *P. knowlesi* genome project was initiated with genomic DNA (gDNA) from the same strain and clone of *P. knowlesi* as the original and subsequent *P. knowlesi* genome projects [see (Pain *et al.* 2008) and Table 1]. This strain was designated as the *H* strain in these projects, and in much of the previous related biological literature. The past and present genome sequences have been based specifically on DNA from the Pk1(A+) clone derived from (Howard *et al.* 1983). However, population genomics research (Assefa *et al.* 2015) has shown that this strain is actually the Malayan strain (American Type Culture Collection Reference #30192/MRA-487), and not the original *H* strain derived from a human infection (Chin *et al.* 1965). It therefore should be referred to as the Malayan strain from this point forward.

**Table 1.** Characteristics of nuclear genome sequences utilized in this study

Name	Genome size (nt)	Scaffold number	Unplaced contig number	Gaps	N50 contig length	N50 scaffold length	Technology
PKNOH-PacBio	24 588 173	N/A	50	N/A	1 207 278	N/A	PacBio
PKNOH-PacBio-Hi-C	24 771 595	14	14	25	16 231	1 832 627	PacBio & Hi-C
PKNH V2	24 359 384	14	148	77	N/A	2 162 603	Sanger & Illumina
PKNA1-C.2	24 359 887	N/A	45	N/A	1 061 780	N/A	PacBio
PKNA1-H.1	23 958 038	N/A	37	N/A	1 017 166	N/A	PacBio

N/A, not applicable.

Genome size includes scaffolds and unplaced contigs. Contigs are only unplaced, *i.e*. non-scaffolded sequences. Gaps are only present in scaffolds. PKNOH data presented here have had organellar sequences removed. Data are from GenBank.

### DNA preparation for PacBio analysis

gDNA was extracted from *ex vivo* matured schizont-stage parasites (Qiagen DNA blood midi kit, Germantown, USA). The gDNA was further purified with a PowerClean DNA cleanup kit (Mo Bio Laboratories, Carlsbad, USA). Five micrograms of gDNA were subsequently used for library preparation. SMRTbell DNA libraries (Pacific Biosciences, Menlo Park, USA) were constructed according to the PacBio standard protocol with BluePippin size selection (Sage Science, Beverly, USA), and sequences were generated on a PacBio RSII instrument using P6-C4 chemistry.

### Reference genome sequences utilized in this study

Several genome sequences were used as comparators (Table 1). Their accession information is as follows: (a) *P. knowlesi*, PKNH (6/18/2015 assembly version 2 and Spring 2017 annotation version) downloaded from PlasmoDB release 32, April 2017 (Aurrecoechea *et al.* 2017); however, the Wellcome Trust Sanger Institute was the original source of the genome sequence and annotation *via* GeneDB (Logan-Klumpler *et al.* 2012); (b) GenBank PKNA1-H.1 whole-genome sequence (WGS) CWHQ00000000.2 and assembly GCA_900005025.2; (c) the genome sequence from the culture-adapted clone PKNA1-C.2 (WGS) CWHR00000000.2 and assembly GCA_900004885.2; and (d) *Plasmodium coatneyi* assembly GCA_001680005.1.

### PacBio analysis and post-generation read processing

After obtaining the raw sequence data from the sequencer (6 PacBio SMRT^®^ cells), the P_filter module was selected to filter the raw reads before applying the HGAP3 assembly algorithm. The P_filter module parameters were minSubReadLength = 500, readScore = 0·80 and minLength = 100. After filtering the reads, HGAP3 *de novo* assembly was performed using the Georgia Advanced Computing Resource Center, GACRC Sapelo cluster SMRT pipeline. The error-correction module was used to quality control the subreads (defined as minimum subread length of 100 bp, a minimum read quality of 0·80) and the longer reads passing this threshold (minimum read length of 6000 bp) were used. The BLASR module was then introduced to align the shorter quality subreads to the seed reads in order to generate corrected consensus reads (Supplementary Table S1). The corrected consensus reads were then assembled into contigs. Contaminating host-derived and organellar contigs were identified by applying BLASTN against the GenBank nr database and they were removed from subsequent analyses. It should be noted that both the mitochondrial and apicoplast genome sequences were recovered as single contigs. Nucmer (Kurtz *et al.* 2004) and SyMAP analyses between the remaining contigs and the *P. knowlesi* reference genome sequence, PKNH (Table 1) were performed to quickly identify regions that were concordant or discordant and to orient our contigs to the established chromosomal orientation (Supplementary Fig. S1). MegaBLAST was used to search the PKNH genome sequence and the results were fed into the Geneious (R9) software package (Kearse *et al.* 2012). The incorporated progressive Mauve algorithm was used for whole genome alignment and visualization.

### Hi-C procedure for P. knowlesi

Synchronized, *P. knowlesi* blood-stage trophozoites were cross-linked in 1·25% formaldehyde for 25 min at 37 °C, quenched in 150 mm glycine for 15 min at 37 °C in a shaking incubator, followed by 15 min on a 4 °C shaking platform and washed in cold phosphate-buffered saline until the pellet is clear of red blood cell debris. Pellets were stored at −80 °C. Parasite pellets were then resuspended in lysis buffer [10 mm Tris-HCl, pH 8·0, 10 mm NaCl, 2 mm 4-(2-aminoethyl)benzenesulfonyl fluoride HCl (AEBSF), 0·25% Igepal CA-360 (v/v), and EDTA-free protease inhibitor cocktail (Roche)] and incubated for 30 min on ice. Nuclei were isolated after homogenization by 15 needle passages. *In situ* Hi-C protocol was performed as described in (Rao *et al.* 2014). Briefly, nuclei were permeabilized using 0·5% sodium dodecyl sulphate. DNA was digested with 100 units of Mbol (NEB, Ipswich, USA), the ends of restriction fragments were filled with biotinylated nucleotides and ligated using T4 DNA ligase (NEB). After reversal of cross-links, ligated DNA was purified and sheared to a length of ~300–500 bp using the Covaris ultrasonicator (S220). Ligated fragments were pulled down using streptavidin beads and prepped for Illumina sequencing by subsequent end-repair, addition of A-overhangs and adapter ligation. Libraries were amplified for a total of 10 PCR cycles [10 cycles of (15 s at 98 °C, 30 s at 55 °C, 30 s at 62 °C)] and sequenced with a NextSeq500 DNA sequencer (Illumina, San Diego, California, USA), generating 75 bp paired-end sequence reads.

### Hi-C data mapping and processing

Approximately 38 million paired-end reads generated by Hi-C assay were mapped to pre- and post-Hi-C PacBio assemblies the HiC-Pro pipeline and its default options (Servant *et al.* 2015). Briefly, this pipeline first attempts to map full-length reads (75 bp in this case) in single-end mode using Bowtie2 with high-sensitivity options ‘*--very-sensitive -L 30 --score-min L,-0·6,-0·2 --end-to-end -reorder*’ (Langmead and Salzberg, 2012). The 17·5–19 m reads mapped for each end at this step. Then, HiC-Pro performs another mapping step where reads with *de novo* Hi-C ligation junctions (GATCGATC in the case of MboI, which cuts from 5′ of each 4-bp GATC pattern) are further trimmed from these junctions and trimmed reads are mapped to the reference genome with similar Bowtie2 settings ‘*--very-sensitive -L 20 --score-min L,-0·6,-0·2 --end-to-end --reorder*’. Another 8·6–9·2 m reads were mapped in this second step making the overall mapping percentage around 80%, which is in line with published, high-quality Hi-C libraries. A large fraction of mapping reads mapped either with no mismatches (~80%) or with a single mismatch (~13%) indicating that our PacBio contigs are of high quality at the base pair level.

Next, mapped single-end reads were paired and filtered out for low-quality mappers (MAPQ score ⩽30), dangling ends, PCR duplicates and other potential assay-specific artefacts to provide a set of valid paired-end reads, which resulted in more than 11 m pairs. These valid pairs were then binned using a fixed-size genomic bin length to create raw/un-normalized inter and intrachromosomal contact maps. The bin sizes ranged from 2 to 50 kb, even though results are only reported for the 10 kb contact maps. These contact maps were then normalized using HiC-Pro's built-in iterative correction method (with default parameters) (Imakaev *et al.* 2012) that is commonly used for eliminating experimental and technical biases in Hi-C contact maps. These normalized contact maps resulting from different versions of the *P. knowlesi* genome assemblies were analysed in order to visualize contact patterns. Raw counts were used at the level of individual restriction enzyme fragments and at 10 kb resolution for correcting problems with the initial set of PacBio contigs, as described below.

### Hi-C correction of the PacBio assembly

Hi-C reads were first mapped to the *de novo* PacBio assembly that consisted of 50 contigs (after removal of six contigs from the initial 56 due to organellar and host contamination) from the nuclear genome with sizes ranging from 5 kb to 2·5 Mb. The resulting intercontig (as opposed to interchromosomal, in the case of complete genomes) Hi-C contact maps indicated that one contig included DNA from three separate non-contiguous loci. Breakpoint junctions for these three regions were identified by finding extremas of the ratio of upstream *vs* downstream interactions from each genomic bin. These junctions were flanked by long stretches of unmappable regions (10–20 kb), which were then extracted as separate contigs. Overall, this ~2·5 Mb contig was broken into five pieces of sizes: 890 kb (mappable) – 20 kb (unmappable) – 490 kb (mappable) – 10 kb (unmappable) – 1100 kb (mappable). This process yielded an assembly of 54 contigs. Hi-C reads were remapped to this new assembly as was done before and the results revealed no evidence of discontinuity in the contact pattern of any contig. This mapping process identified 15 short contigs, ranging from 4 to 22 kb, which did not have a substantial number of contacts with any other contig and, hence, could not be placed into any scaffold. Next, clustering on intercontig contact maps of the 39 remaining contigs yielded 14 clusters that putatively correspond to the 14 different chromosomes/scaffolds of the PKNOH genome assembled in this work (see Supplementary Table S2). Our clustering was guided by initial assignments of contigs to chromosomes from the previous PKNH assembly. Specifically, each of our 14 clusters was initialized with contigs that are assigned to the same chromosome in the PKNH assembly (see Supplementary Table 2 for the initial naming of contigs that included putative chromosome assignments). Then, a refinement step where any contig pair with multiple interactions of at least 20× higher than the expected interchromosomal count was placed in the same cluster and this process was repeated until no such intercontig interactions existed among contigs from different clusters. For each cluster that contained more than one contig, the order and orientation of each of these contigs with respect to each other was determined using Hi-C contact patterns to yield a single, contiguous scaffold. This process began with the pair of contig ends that have the highest number of Hi-C interactions with each other and iteratively decreased the number of contigs by one in each step until only one scaffold was left. For example, for chromosome 12 with two contigs (say cA, cB) out of 39, the number of interactions spanning the junctions were computed for all four combination possibilities cA with cB, namely (cA-rightEnd – cB-rightEnd), (cA-rightEnd – cB-leftEnd), (cA-leftEnd – cB-rightEnd) and (cA-leftEnd – cB-leftEnd), and the highest scoring configuration was picked as the final scaffold. A similar idea applies to chromosomes with more than two contigs. As a *post hoc* validation of the resulting Hi-C corrected assembly, all Hi-C data were remapped back to the new PacBio reference genome and analysed to show that: (i) all intrachromosomal interactions follow the expected genomic distance scaling (Ay *et al.* 2014a), (ii) there are no interchromosomal interactions that are at the level of interactions between a pair of proximal regions on the same chromosome and (iii) the level of interaction between two newly joined contigs is similar to that of two sides of a random contiguous region.

### Genome annotation

Two different annotation approaches were used. First, *ab initio* gene predictions were produced using the SNAP (Korf, 2004) and Augustus (Stanke *et al.* 2008) algorithms as implemented in the MAKER2 (Holt and Yandell, 2011) genome annotation and data management tool. Second, the assembled genome sequence was submitted to the Companion Web server (Steinbiss *et al.* 2016) for annotation and analysis of parasite genomes using a reference-based approach. In both cases, the proteome reference was from the same strain: *P. knowlesi* H (PKNH, annotation obtained from PlasmoDB annotation version 6-18-2015). Evidence-based gene annotation in MAKER2 was produced using default settings. The genome annotation in Companion was produced using the default settings, with the following two exceptions: the ‘do not modify my input sequence’ option was selected, and the AUGUSTUS score threshold was set to 0·5.

To evaluate the gene models produced by the MAKER2 and Companion approaches, a comparison between both annotations was performed using ParsEval (Standage and Brendel, 2012). The unique genes identified by each approach were selected for manual curation using Artemis (Rutherford *et al.* 2000). To assign functional annotation to the predicted proteins, the information generated by Companion was kept, and for annotated proteins that were not predicted by Companion, searches for orthologs using OrthoFinder (Emms and Kelly, 2015) were performed and the functional information obtained was transferred to the final annotation. InterProScan5 (Jones *et al.* 2014) was also used to help assign a putative function to proteins. Tandem Repeat Finder (Benson, 1999) was used to identify nucleotide repeats. Manual annotation was done using Artemis version 16.0.

### Comparisons to existing P. knowlesi genome assemblies and annotation

Predicted protein-encoding genes identified in the PKNOH-manually curated annotation were compared with the existing annotations of PKNH and PKNA1-H.1 (see Table 1). The comparisons were performed using OrthoFinder (Emms and Kelly, 2015) with default parameter settings. The PKNOH-manually annotated protein-encoding gene sequences were compared with a recently released PKNH April 2017 annotation using BLASTp and looking for reciprocal best hits as well as no ‘hit’, *i.e*. unique predictions in either annotation. A protein domain analysis was also conducted to characterize any differences. A comparison of predicted protein lengths was also performed using custom PERL scripts.

## RESULTS

A refined *P. knowlesi* genome sequence has been generated *de novo* from gDNA of the Pk1(A+) clone of the Malayan strain of this species (see Materials and Methods). Both nuclear and organellar sequences were assembled from PacBio reads. PacBio unitigs were verified, ordered and oriented into scaffolds with the assistance of Hi-C. This new genome sequence, named here as the ‘MaHPIC Pk genome sequence’, can be accessed *via* GenBank Assembly accession number: GCA_002140095.1. This sequence is associated with the MaHPIC NCBI Umbrella BioProject: PRJNA368917 and PKNOH BioProject: PRJNA377737. This Whole Genome Shotgun project has been deposited at DDBJ/ENA/GenBank under the accession NETL00000000, which is the version described in this paper. The organellar genome sequences have accession numbers: MF373419 & MF373420. The SRA accessions are: SRR5413221 (Hi-C Illumina) and accessions SRR5413213-SRR5413218 (PacBio).

### The P. knowlesi PacBio and Pk-PacBio-HiC assemblies

The Pk-PacBio assembly was created as follows. PacBio sequence generation and subread assembly resulted in the generation of 56 polished contigs (Supplementary Table S1). Seven of these contigs represent organellar genome or host contaminant sequences, leaving a total of 49 contigs representative of the nuclear genome, 35 of which have a read depth of >50. These 35 contigs have a combined length of 24 579 173 bp with the largest contig being 2 476 829 bp. A SyMAP dotpot analysis was performed to assess the coverage, orientation and relationship of the Pk-PacBio assembly contigs to the existing *P. knowlesi* PKNH version 2 sequence (Table 1). The analysis revealed that there was not always a 1 : 1 correspondence. Overall, significant synteny was observed, but some PacBio contigs aligned with multiple chromosomes of the PKNH genome sequence and some contigs needed their sequence orientation flipped to match the established orientations for *P. knowlesi* PKNH chromosomes (Supplementary Fig. S1). Since genome sequences can misassemble at repetitive sequence regions, additional validation was sought with a Hi-C analysis of intrachromosomal interactions.

Hi-C analyses generate genome-wide chromatin interaction datasets to probe the three-dimensional architecture of chromosomes within the nucleus. Hi-C relies on the frequency of contact between all pairs of loci in the genome. These data also provide valuable information for contiguity since two loci that are nearby in the linear genome interact at a higher level on average. This property has been successfully utilized in assembling, ordering and orienting genomic sequences into chromosomes (Korbel and Lee, 2013; Flot *et al.* 2015; Bickhart *et al.* 2017; Dudchenko *et al.* 2017). This technology was applied here for *P*. *knowlesi* parasites of the Malayan strain Pk1(A+) clone, using proximity ligation and massively parallel sequencing, with interacting genomic regions captured *via* paired-end reads on the Illumina sequencing platform. Computational analysis of the Hi-C data revealed that 34 of the 35 high-coverage contigs (PacBio >50×) together with three of the 14 low-coverage contigs (<50× PacBio) have Hi-C reads that map to them.

**Fig. 1.**
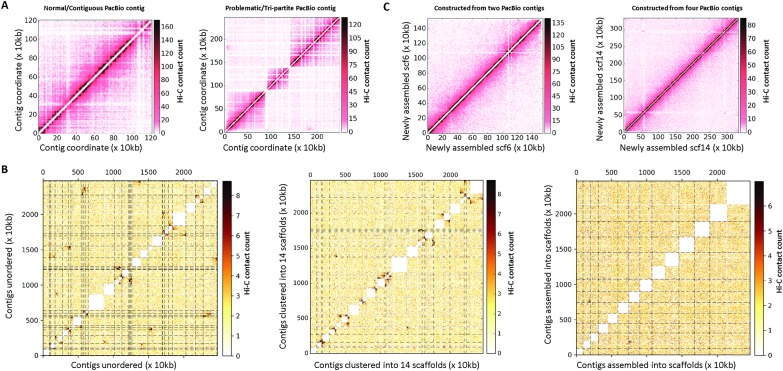
Hi-C assisted scaffolding of PacBio contigs. (A) Alignment of Hi-C data to the initial set of 35 high-coverage contigs by PacBio assembly showed that one of the contigs includes DNA from three different chromosomes as evidenced by the tri-partite structure of intracontig contact map of this contig (right). Other contigs did not exhibit similar contact patterns (representative example – left) suggesting they are contiguous pieces from a single chromosome. (B) Intercontig Hi-C contact maps of the unordered set of contigs (left) that were named according to their similarity with chromosomes in the PKNH assembly show striking off-diagonal contact enrichment suggesting that pairs of contigs that belong to the same chromosome are not ordered consecutively. Similar intercontig maps when contigs are clustered into scaffolds according to their Hi-C contact counts (mid) show minimal off-diagonal enrichment. Interchromosomal/scaffold contact map generated by aligning Hi-C reads to the new, chromosome level assembly (right) exhibits contact patterns that are expected of and observed in *Plasmodium* and yeast species (Ay *et al.* 2014b; Duan *et al.* 2010). This assembly was generated by breaking down the problematic contig, clustering contigs into chromosomal groups, and ordering and reorienting contigs within each group to maximize Hi-C contacts between adjacent and correctly oriented contigs to create scaffolds representative of each chromosome. (C) Intrascaffold Hi-C contact maps (normalized counts, 10 kb resolution) from two representative scaffolds in the new assembly. Scaffold 6 (left) and scaffold 14 were constructed by joining two and four PacBio contigs, respectively. The rows/columns marked by white represent unmappable or poorly mappable regions with Hi-C reads (Illumina 76 × 2 bp, paired-end sequencing).

Of the high-coverage contigs, the largest, (contig 0), had three regions that showed segregated (i.e. tripartite) interaction patterns suggestive of a PacBio misassembly (Fig. 1A). This contig was split into three large contigs and two small contigs (one 10 kb and one 20 kb) at the boundaries that were not mappable with Hi-C reads. With the addition of these three new contigs, excluding the two that are unmappable, a total of 39 contigs could be confidently ordered and oriented into 14 scaffolds with a total of only 25 gaps (Fig. 1, Supplementary Table S2). The final assembly, called Pk-PacBio-HiC, contains 14 scaffolds (assembled from 36 high-coverage and three lower-coverage contigs) with an N50 of 1 832 627 bp and 14 unplaced contigs. The consensus genome sequence for this assembly has the GenBank Locus Tag PKNOH (Table 1) and is referred to as the MaHPIC Pk genome sequence. The 14 unplaced contigs have an N50 of 16 231 bp and a total length of ~184 kb. To further examine their gene content a BLASTX analysis was performed. Most of these 14 unplaced contigs contain *SICAvar* gene fragments, and a few pseudogenes but no additional full-length genes (data not shown).

### *MaHPIC Pk genome sequence PKNOH comparison with the PKNH version*2 *sequence*

**Fig. 2.**
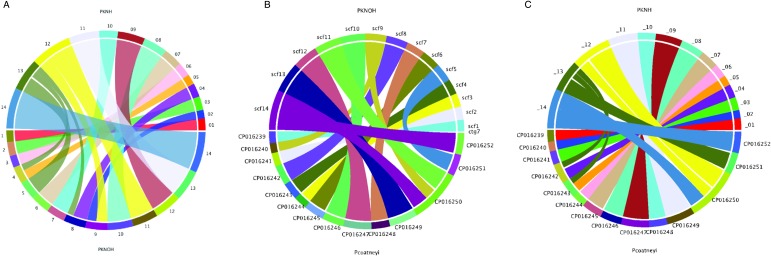
Chromosomal synteny between PKNH and the MaHPIC PKNOH genome sequences. (A) SyMAP circular DNA comparison of the MaHPIC Pk genome sequence scaffolds to the PKNH 2015 consensus sequence. (B) SyMAP circular DNA comparison of the MaHPIC Pk genome sequence scaffolds to the *Plasmodium coatneyi* HACKERI genome sequence that was assembled using PacBio technologies (Chien *et al.* 2016). (C) SyMAP circular DNA comparison of the PKNH 2015 consensus sequence and *P. coatneyi* genome sequence.

Following the correction of the Pk-PacBio assembly with the addition of Hi-C, a synteny analysis was performed between the resulting genome sequence and the existing PKNH version 2 genome sequence using SyMAP (version 4.2) (Soderlund *et al.* 2011). Ten of the PKNOH scaffolds (1, 2, 3, 6, 7, 10, 11, 12, 13 and 14) are fully syntenic with chromosomes in the PKNH assembly attesting to the quality of both assemblies (Fig. 2A). However, the Hi-C validated genome sequence also contains a few rearrangements of chromosomal blocks with respect to PKNH version 2. Portions of MaHPIC PKNOH scaffolds 4 and 8 as well as all of scaffold 5 map to the current PKNH chromosome 13. The remainder of the MaHPIC PKNOH scaffold 4 maps to the entirety of PKNH chromosome 5 and the remainder of MaHPIC PKNOH scaffold 8 maps to all of PKNH chromosome 4. MaHPIC PKNOH scaffold 9 contains all of chromosome 2 and a portion of chromosome 12 (Fig. 2A and B).

**Fig. 3.**
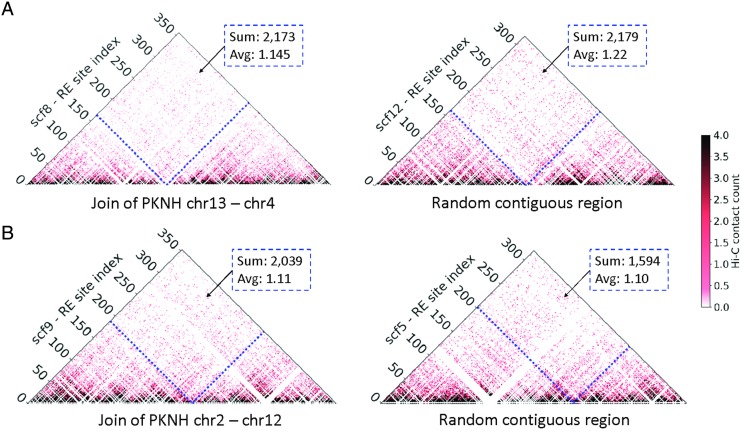
Hi-C contact maps for the join regions present on scaffolds 8 and 9. Hi-C contact maps of two scaffolds from the PKNOH-PacBio-Hi-C assembly that contain contigs previously assigned to two different chromosomes in the PKNH assembly. These contact maps are zoomed in to the join regions and are at the single MboI restriction fragment level (~1 kb in resolution). Each heatmap is rotated 45 degrees compared with previous intracontig/scaffold heatmaps for visualization purposes. (A) The 200 kb region of scaffold 8 (scf8:500 000– 700 000) that surrounds the join (at scf8:593 400) between two contigs previously assigned to chr13 and chr4 (left) compared with a matched 200 kb region from scaffold 12, which consists of a single contiguous PacBio contig (right). (B) Similar case *vs* control figure for scaffold 9 compared with matched coordinates in scaffold 5. The dashed blue lines correspond to location of the join (or matching coordinates on the right) and the sum and average number (excluding zeros) of interactions between the left and right (rectangular area) of a join are reported for each case.

To validate these findings, two analyses were performed. First, an analysis of the Hi-C data at these novel fusion junctions was performed. Fig. 3A shows the heatmap for a 200 kb region on scaffold 8 that corresponds to the join of sequences assigned to chromosomes 13 and 4 in the PKNH version 2 genome sequence. Hi-C analysis supports the PKNOH arrangement of sequences. Figure 3B shows similar support for the novel fusion on scaffold 9. Second, PacBio coverage across these novel fusion junctions was also assessed (Supplementary Fig. S3A–C). Each of the regions examined has PacBio coverage to a depth of ~249–279. As this depth is higher than the 151× average coverage for the genome, the data suggest that this region is repetitive and may itself be compressed in the MaHPIC Pk genome sequence.

While the PacBio sequence provides support for the novel fusions relative to PKNH, it was not able to span some gaps that Hi-C was able to span (Supplementary Fig. S4A). PacBio was, however, able to close a gap that is present in the PKNH version 2 genome sequence (Supplementary Fig. S4B). There is one section of PKNH chromosome 13 (nucleotides 1 125 179–1 177 288) that does not have any obvious synteny with the MaHPIC PKNOH genome sequence (Fig. 2A). This 52 kb sequence contains many short repetitive nucleotide sequences (Supplementary Fig. S5) and shares significant similarity with ~30 kb of PKNH chromosome 5 as well (data not shown). A BLASTX analysis of this region shows that it encodes portions of SICA proteins. Importantly, a synteny analysis of the MaHPIC Pk genome sequence with the genome sequence of the closely related *P. coatneyi* species (Chien *et al.* 2016) revealed that both species are syntenic for the region equivalent to PKNH chromosome 13 (each is missing this 52 kb region), and that overall, these two species have fewer rearrangements relative to each other than either has with the PKNH genome sequence (Fig. 2B and C).

Overall, the MaHPIC Pk genome sequence, at 24·59 Mb is the longest *P. knowlesi* sequence generated to date, with an additional ~650 kb of sequence assembled (Table 1). It also has the fewest number of gaps with only 25 remaining. While the N50 for MaHPIC Pk genome sequence (PKNOH) is slightly smaller than the PKNH genome sequence, it does contain the largest scaffold created to date at 3·31 Mb. Importantly, the new genome sequence has extended the subtelomeric regions of the chromosomes by ~20 kb. Using Tandem Repeats Finder, telomeric regions were analysed where additional sequence was added to chromosomes. There are REP20-like 21–25 bp repeats located on eight scaffolds in eight regions (left or right subtelomere) ranging from 40 copies on scaffold 2 up to 165 copies on scaffold 11. Some telomeres share consensus repeat sequences, such as scf1(R), scf7(R) and scf10(R). Typical *Plasmodium* telomere repeats were identified on eight scaffolds, and scaffold 6 has telomere repeats on both ends (Supplementary Table S3).

### MaHPIC Pk genome sequence annotation and gene content

From the combined automated and manual annotation, the MaHPIC Pk genome sequence is predicted to contain 5217 protein-encoding genes, 45 tRNAs and 12 rRNA clusters. The PKNOH annotation has 2331 uncharacterized, i.e. hypothetical proteins, whereas the updated PKNH has only 2081 that are categorized into type ‘unknown’ and further classified based on conservation and predicted secretion status. In total, 4775 of the PKNOH protein-encoding genes are orthologous to PKNH (version 2) and PKNA1-H annotated proteins (Table 2) (Supplementary Fig. S6) based on OrthoFinder and 5135 of the PKNOH and PKNH proteins are reciprocal best BLASTp hits of each other, with 4890 being identical in both length and sequence (Supplementary Table S4) indicating a high level of congruence between the two annotation efforts. The PKNOH annotation contains 82 unique protein-encoding genes, 24 of which are *SICAvars* and the PKNH annotation contains 148 unique protein-encoding annotations, 100 of which are *SICAvars* (mainly fragments) (Supplementary Tables S5 and S6). To examine the effect of the more complete assembly on the annotation, the average protein length between PKNOH and PKNH was examined. Overall there was no significant change (see Supplementary Fig. S7). It is interesting to note that on average, predicted *P. knowlesi* proteins are shorter, at 476aa, than *P. coatneyi* Hackeri at 699aa, *Plasmodium cynomolgi* B strain at 575aa, or *Plasmodium falciparum* 3D7 at 766aa.

**Table 2.** Nuclear genome annotation metrics

Name	Genes	Proteins	tRNA	rRNA	Pseudogenes
PKNH	5483	5282	45	11	8
PKNA1-H.1	5373	5138	45	14	4
PKNOH-Maker2	5356	5300	45	11	N/A
PKNOH-Companion	5315	5253	45	11	152
PKNOH-manually curated	5342	5217	45	12	22

N/A, not applicable.

Values obtained from archival records or generated in this study; see ‘Materials and Methods’ section.

### SICAvar multigene family manual annotation and pseudogene analyses

**Fig. 4.**
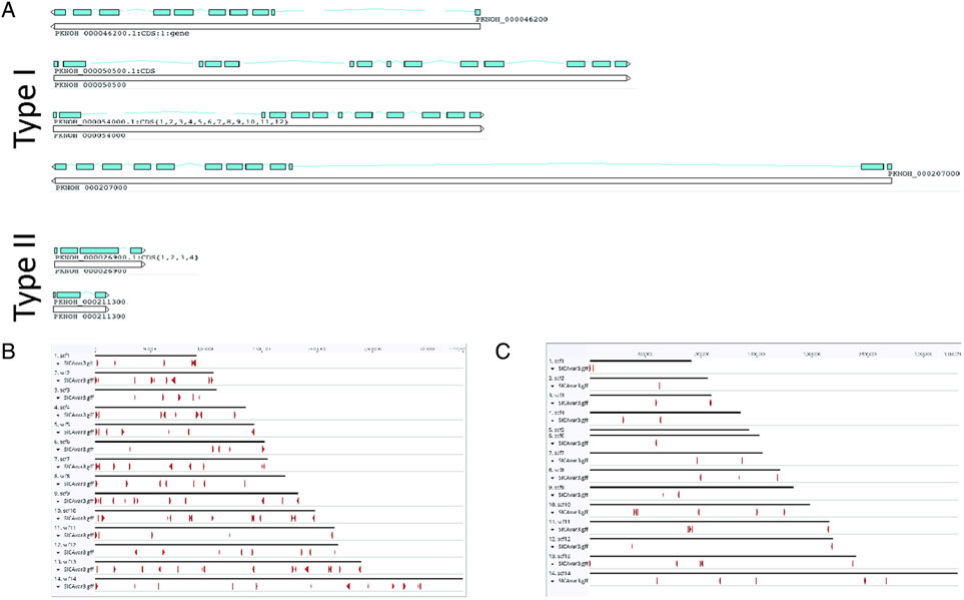
SICAvar distribution and gene models. (A) Shown are representative examples of types I and II SICAvar genes with exons noted in blue, and their directionality indicated with arrow heads placed at the end of the 3-prime exons. Type I SICAvar genes are characterized by multiple exons (5–16), often with extremely large introns, particularly between exons 2 and 3. Type II SICAvar genes have three or four exons and are more compact with smaller introns. In five of the six examples shown, the initial two exons shown are typical. (B) Distribution of full SICAvar genes (types I and II) along the PKNOH scaffolds. (C) Distribution of partial SICAvar gene segments (types I and II) along the PKNOH scaffolds.

As a final step to ensure the most useful product for ongoing genetic and biological studies, a manual annotation of the large and complex *SICAvar* multigene family was performed on the scaffolds and unplaced contigs and pseudogenes were checked and manually corrected. The use of PacBio has led to major improvements in structure and placement of the multi-exon *SICAvar* genes; confirming the genes with up to 16 exons; 5–16 exons in type I *SICAvar* genes, and three or four exons in type II *SICAvar* genes. A summary of the current *SICAvar* gene repertoire is in Table 3 and Fig. 4A shows how they are dispersed throughout the entire genome sequence. Many incomplete *SICAvar* gene sequences, noted before as fragments, are now complete as full-length genes (Table 3, Fig. 4B). In all, over 50 manual corrections to *SICAvar* gene models were completed, resulting in an additional 30 full-length type I *SICAvar* genes, resulting in a total of 136 complete genes; 117 type I and 19 type II. The corrections to gene models included: resolving gaps with the addition of PacBio sequence; the identification of additional exons located within gaps and subtelomeric regions; and correctly connecting portions of genes that were previously assigned to incorrect chromosomes. Using InterProScan searches, the number of SICA domains was compared between the PKNH and MaHPIC Pk assemblies (Table 3). The total number of extracellular *α* and *β* domains and inner membrane C-terminal domains between the two assemblies is similar indicating that the improved *SICAvar* annotation reported here is the result of resolving gaps and better overall organization of the genome. Importantly, most full-length *SICAvar* genes, whether type I or II, have a characteristic initial first two exons where a PEXEL motif has been noted (Lapp *et al.* 2009), and final two exons encoding a transmembrane domain and conserved cytoplasmic domain (al-Khedery *et al.* 1999).

**Table 3.** Comparative *SICAvar* gene statistics in the PKNH (April 2017) and PKNOH (MAHPIC PacBio) assemblies

	PKNOH		PKNH	
	Type I	Type II	Type I	Type II
Full *SICAvar* genes	117	19	87	14
*SICAvar* fragments	22	0	128	5
Exon #	11 (5–16)	3 (3–4)	11 (3–16)	3 (3–4)
Gene length (nt)	14 159 (6312–32 900)	3473 (2051–4269)	ND	ND
Coding length	5652 (834–6777)	1488 (963–3051)	5940 (1401–8295)	2630 (1302–3309)
Protein length (aa)	1892 (278–2783)	496 (321–1017)	1979 (466–2764)	876 (433–1102)
Alpha domains	133		138	
Beta domains	763		765	
*C*-terminal domains	133		143	

Exon number and lengths are reported as median values of the ranges provided. Protein domain counts include full *SICAvar* proteins and fragments of both types.

Importantly, 134 of 152 pseudogenes identified during the genome annotation process were manually adjusted. In many cases, predicted pseudogenes involved a frameshift that occurred *via* the addition of DNA sequence compared with the PKNH sequence. In most cases, predicted splice sites could be identified to resolve these. Notably, other predicted pseudogenes match their PKNH counterparts exactly, but were assigned as pseudogenes; these often included small homopolymeric ‘A’ intronic sequences not annotated in the PKNH reference genome (Supplementary Fig. S8).

## DISCUSSION

The MaHPIC *P. knowlesi* genome sequence, presented here, is the most advanced *P. knowlesi* genome assembly and sequence to date with regards the methods used, and the degree of confirmatory validation undertaken to ensure the accurate contiguity of the sequences and gene annotations. Recently, a PacBio-based *de novo* genome sequence was published for the closely related species, *P. coatneyi* (Chien *et al.* 2016) and the MaHPIC Pk genome sequence was initiated with similar procedures. The *P. coatneyi* genome assembly achievement demonstrated the ability of PacBio to assemble complicated repetitive regions by taking advantage of the superior long-read capabilities of this technology (Chien *et al.* 2016). However, it is now clear that this technology alone does not guarantee correct contig scaffolding (ordering and orientation) and gap closure; thus, additional steps and experimental data are required for a correct assembly (Hunt *et al.* 2014). The combination of PacBio and Hi-C technologies have provided the great benefit of enabling the confirmation of the current assembly, with a high level of confidence in the placement and ordering of the contig sequences. Then, a combination of automated annotation using Maker (Holt and Yandell, 2011) and Companion (Steinbiss *et al.* 2016), followed by manual annotation provided a high degree of confidence in gene calling and correction of 134 genes that had been considered pseudogenes.

The assembly, confirmatory and annotation approaches used have yielded the largest *P. knowlesi* genome sequence to date by more than 410 kb and the largest single scaffold at 3·31 Mb. Much of the sequence length was gained in subtelomeric regions where many repetitive sequences are located. Importantly, this project provides an example of a relatively rapid and economical assembly and annotation, by a small team of experts, thus showcasing the broad value of the latest technological advances. The MaHPIC *P. knowlesi* nuclear genome sequence is presented as 24·77 Mb on 14 scaffolds and 14 contigs with only 25 gaps (down from 190 and 77 gaps reported in the previous genome sequences, which also had as many as 148 unplaced contigs compared with 14 now, see Table 1).

Long-read PacBio sequences and the HGAP assembly process were important to obtain high-quality contigs that could traverse many of the genome's repetitive sequences. However, despite the superior length of PacBio reads, ranging from 2500 bp to >35 kb, Hi-C proved to be essential for the correct scaffolding of PacBio contigs and the resolution of one misassembled contig. Specifically, the HGAP algorithm misassembled contig0, the largest contig generated at ~2·5 Mb. Hi-C chromatin interaction profile data revealed this misassembly and suggested regions where the misassembled contig should be divided; repetitive sequences were associated with the misassembly of this contig. The Hi-C methodology was developed to study the three-dimensional folding of the genome as well as the physical interactions that link regulatory elements with distant sequences. Importantly, as demonstrated here, this methodology can provide a sophisticated solution to improve chromosome-scale assemblies and accurately position individual contigs without a requirement for sequence overlap (Kaplan and Dekker, 2013; Korbel and Lee, 2013; Flot *et al.* 2015; Bickhart *et al.* 2017; Dudchenko *et al.* 2017).

The current high-quality *P. knowlesi* genome sequence assembly and annotation are critical for gaining an improved understanding of *P. knowlesi* biology, genetic mechanisms and evolution. The PKNOH and PKNH annotation are highly similar with 4890 identical gene structures and sequences attesting to the congruence of the two annotation approaches. There are 82 unique PKNOH genes and 148 unique PKNH genes as well as 244 PKNOH genes that share significant regions of identity with PKNH genes. The majority, but not all, of the difference are focused on the *SICAvar* gene family.

*Plasmodium knowlesi* is a model parasite for studies of antigenic variation in malaria, including *P. falciparum* malaria, reviewed in (Galinski *et al.* 2017) in this special issue. Special emphasis was placed on the *SICAvar* multigene family because of its known importance in investigating *Plasmodium* immune evasion strategies, but also given the team's goal to successfully tackle a number of genomic level challenges with the originally estimated 108 *SICAvar* genes and their complexity with as many as 16 exons reported, long repeat-laden introns (al-Khedery *et al.* 1999; Corredor *et al.* 2004; Pain *et al.* 2008; Lapp *et al.* 2009), and complex repeat patterns in the 3′ untranslated region (UTR) sequences that may have functional implications in post-transcriptional regulatory processes (Corredor *et al.* 2004). The original *P. knowlesi* genome sequence annotation from 2008 described 29 full-length *SICAvar* genes, with designations as type I for the longer genes and type II for shorter genes (Pain *et al.* 2008), and online updates to the PKNH annotation (GeneDB Pk version 2, April 2017) have described 101 full-length *SICAvar* genes to date (87 type I and 14 type II). Here, 136 full-length *SICAvar* genes have been identified and annotated: 117 type I and 19 type II. The type I genes can span as much as 33 kb and contain roughly 6–7 kb of coding sequence. The longest *SICAvar* intron is 23 kb, present between exons 2 and 3 of gene PKNOH_S130207000.1. These type I *SICAvar* introns are considerably long relative to most other genes in the genome. Moreover, while more than half of *P. knowlesi* genes have introns, the absolute majority has only one intron. The presence of these challenging repeat-laden sequences is a main reason why it has been difficult to accurately piece together this genome and this particular gene family using short-read sequencing technologies. Interestingly as well, *SICAvar* introns begin at the start of cysteine-rich encoding domains, and their structure suggests that exon shuffling is a possible means of generating diversity within a population (al-Khedery *et al.* 1999). Finally, the original genome publication had questioned if some observed *SICAvar* ‘fragments’ were real or if they reflected sequence problems. Twenty-two *SICAvar* fragments remain, and many contain either the first two upstream exons or the last three exons (including, the penultimate TMD and final exon-encoding cytoplasmic domain sequences). Some of these are bonafide fragments, since they occur immediately next to or between other genes. These are possibly remnants from recombination events in their evolutionary history, or they may function in recombinatorial events associated with *in vivo* switch mechanisms as shown previously to have occurred at the 3′ region (al-Khedery *et al.* 1999). Additional research on *in vivo* switch events will help to determine the frequency with which they may occur. In any case, questions need to be addressed regarding the functional roles these sequences may play, including as possible templates for non-coding RNAs (Pain *et al.* 2008; Galinski *et al.* 2017). The MaHPIC Pk genome sequence obtained in this study will support future analyses of gene expression, including investigations of the regulation of the *SICAvar* multigene family (Galinski *et al.* 2017), and specifically lead to a better understanding of the complex genetic and epigenetic processes used by *P. knowlesi* throughout its developmental life cycle and to evade the host's immune system. The ability to study the regulation of the *SICAvar* and other gene families is now possible, with greater confidence, including epigenetic processes. To conduct such research, it is critical to know and monitor the expression of the correct full-length gene sequences, and be able to evaluate the functions of the 5′ and 3′ UTRs, intron sequences and antisense non-coding RNAs. Correct assembly of these sequences and their confirmed placements on the scaffolds are also critical to determining if recombination events are associated with switches in gene expression. The originally defined *SICAvar* recombination event associated with an *in vivo* switch in *SICAvar* expression involved movement of *SICAvar* sequence from one chromosome to the end of the expressed 205 kDa *SICAvar* gene on a different chromosome (Corredor *et al.* 2004). Moreover, the new MaHPIC reference genome sequence will support increased accuracy of data generated in quantitative gene expression analyses that are critical for understanding the parasite's regulatory mechanisms and how these may change in the context of altered host or culture environments. For example, the molecular mechanisms that regulate expression of the *SICAvar* genes are dependent on the host environment, including unknown splenic factors (Barnwell *et al.* 1982, 1983; Lapp *et al.* 2013). The expression of the *SICAvar* genes is dramatically downregulated in splenectomized rhesus macaques, as well as in *in vitro* culture (Lapp *et al.* 2015). In this context, it will be of interest to determine what other genes are co-regulated as *SICAvar* gene expression is upregulated or downregulated, as members of this gene family are turned on or off.

### Concluding remarks

In summary, the MaHPIC Pk genome sequence is the most complete *P. knowlesi* genome sequence generated to date, and this can aid basic research on various topics that can include biological and immunological mechanisms relating to multigene families and antigenic variation, but also vaccine and drug candidate discovery. This sequence, generated using PacBio and Hi-C technologies, demonstrates the superiority of the combined technologies for rapidly and economically generating more complete *de novo* assemblies of difficult-to-sequence genomes including better resolution of repetitive sequences. Together, both technologies helped to distinguish the correct assembly of the genome's large scaffolds from assembly artefacts and the subtelomeric regions were extended with confidence. The methods used here will facilitate comparative and functional genomics analysis of this parasite as needed for other laboratory strains, isolates or clones, and for population biology studies.

## Supplementary material

For supplementary material accompanying this paper visit http://dx.doi.org/10.1017/S0031182017001329. click here to view supplementary material
